# The Effects of Mind-Body Therapies on the Immune System: Meta-Analysis

**DOI:** 10.1371/journal.pone.0100903

**Published:** 2014-07-02

**Authors:** Nani Morgan, Michael R. Irwin, Mei Chung, Chenchen Wang

**Affiliations:** 1 Center for Integrative Medicine, Division of Rheumatology, Tufts Medical Center, Tufts University School of Medicine, Boston, Massachusetts, United States of America; 2 Cousins Center for Psychoneuroimmunology, UCLA Semel Institute for Neuroscience, University of California Los Angeles, Los Angeles, California, United States of America; 3 Nutrition/Infection Unit, Department of Public Health and Community Medicine, Tufts University School of Medicine, Boston, Massachusetts, United States of America; University of Sao Paulo, Brazil

## Abstract

**Importance:**

Psychological and health-restorative benefits of mind-body therapies have been investigated, but their impact on the immune system remain less defined.

**Objective:**

To conduct the first comprehensive review of available controlled trial evidence to evaluate the effects of mind-body therapies on the immune system, focusing on markers of inflammation and anti-viral related immune responses.

**Methods:**

Data sources included MEDLINE, CINAHL, SPORTDiscus, and PsycINFO through September 1, 2013. Randomized controlled trials published in English evaluating at least four weeks of Tai Chi, Qi Gong, meditation, or Yoga that reported immune outcome measures were selected. Studies were synthesized separately by inflammatory (n = 18), anti-viral related immunity (n = 7), and enumerative (n = 14) outcomes measures. We performed random-effects meta-analyses using standardized mean difference when appropriate.

**Results:**

Thirty-four studies published in 39 articles (total 2, 219 participants) met inclusion criteria. For inflammatory measures, after 7 to 16 weeks of mind-body intervention, there was a moderate effect on reduction of C-reactive protein (effect size [ES], 0.58; 95% confidence interval [CI], 0.04 to 1.12), a small but not statistically significant reduction of interleukin-6 (ES, 0.35; 95% CI, −0.04 to 0.75), and negligible effect on tumor necrosis factor-α (ES, 0.21; 95% CI, −0.15 to 0.58). For anti-viral related immune and enumerative measures, there were negligible effects on CD4 counts (ES, 0.15; 95% CI, −0.04 to 0.34) and natural killer cell counts (ES, 0.12, 95% CI −0.21 to 0.45). Some evidence indicated mind-body therapies increase immune responses to vaccination.

**Conclusions:**

Mind-body therapies reduce markers of inflammation and influence virus-specific immune responses to vaccination despite minimal evidence suggesting effects on resting anti-viral or enumerative measures. These immunomodulatory effects, albeit incomplete, warrant further methodologically rigorous studies to determine the clinical implications of these findings for inflammatory and infectious disease outcomes.

## Introduction

Over the last two decades, mind-body therapies (MBTs), including Tai Chi, Qi Gong, meditation, and Yoga have received increasing awareness and attention from the scientific community seeking to understand the safety and efficacy of these widely used practices. According to the 2007 National Health Interview Survey, 19% of American adults have used at least one mind-body therapy in the past 12 months [1]. Currently, the National Center for Complementary and Alternative Medicine designates MBTs as a top research priority [2].

Previous work has shown that MBTs offer many psychological and health functioning benefits including reductions in disease symptoms, improvements in coping, behavior regulation, quality of life, and well-being [3]–[22]. In light of these benefits, recent investigations have sought to better understand the role of MBTs on physiological pathways such as the immune system. It has been well-established that psychological stress and depression impair anti-viral immune responses and activate innate immunity or markers of inflammation via effector pathways, such as the sympathetic nervous system and the hypothalamus-pituitary-adrenal (HPA) axis [23]–[28]. In fact, behavioral interventions targeted at alleviating stress, promoting heightened states of relaxation, and encouraging moderate physical activity, have been shown to bolster anti-viral immune responses and decrease markers of inflammation, particularly among older adults or adults experiencing high levels of psychological stress [3], [17], [18], [29]–[31].

The efficacy of such behavioral interventions in modulating the immune system suggests that MBTs may also confer immunomodulatory benefits. Tai Chi, Qi Gong, and Yoga are multi-dimensional behavioral therapies that integrate moderate physical activity, deep breathing, and meditation to promote stress-reduction and relaxation, which could potentially influence the immune system [28]. Meditation, including more integrative, mindfulness**-**based, stress-reduction programs, has also been shown to regulate emotional and affective responses to stress, and therefore may influence the immune system even in the absence of physical activity [32].

To our knowledge, this study is the first comprehensive review of the best available evidence, summarizing the effects of MBTs on the immune system while focusing on two aspects of immunity that are regulated by stress response mechanisms, namely inflammation and anti-viral related immune responses [28]. Based on these findings, recommendations for future research are offered.

## Methods

### Data Sources and Searches

We searched MEDLINE (from 1946), CINAHL (from 1981), SPORTDiscus (from 1985), and PsycINFO (from 1967) through September 1, 2013. Searches were limited to human studies and the English language (the full search strategy is described in **Table S1**). We also screened the reference lists of selected reviews and primary articles for additional publications and consulted experts in the field. We did not search for unpublished studies.

### Study Selection and Eligibility Criteria

All abstracts identified through the literature search were screened with a low threshold to exclude irrelevant abstracts. Full-text articles of potentially relevant abstracts were retrieved and evaluated for eligibility by one investigator and confirmed by another investigator. Study eligibility criteria are described in **Table 1**.

**Table 1:** Study Eligibility Criteria.

Study Design	Published data from randomized controlled trials
Population	Adults, clinical or research populations
Intervention	Tai Chi, Qi Gong, meditation, or Yoga interventions, or as these mind-body therapies as a major component of the intervention, with duration of four weeks or longer. In studies that involved more than one active intervention, we restricted our analyses to the comparisons between mind-body therapy intervention and control group. For meta-analysis, we used the time-point immediately following completion of the intervention if multiple time-points were measured in a study
Comparator	Any control
Outcome	At least one immune outcome
Minimal sample size	10 participants

Classification of the immune outcomes for this meta-analysis was guided by functional genomics studies that have identified two broad gene expression programs that can be induced in myeloid lineage cells by different types of microbial stimulus, and that can also be regulated by stress response mechanisms [28]. For example, extracellular pathogens, such as bacteria, activate transcription factors (i.e., nuclear factor-κB (NF-κB) and activator protein 1 (AP1), which lead to increased expression of genes such as interleukin-1β (*IL-1B*), interleukin-6 (*IL-6),* and tumor necrosis factor (*TNF*). Together, activation of these inflammatory gene programs leads to increases in cellular markers of inflammation such as the pro-inflammatory cytokines IL-6, TNFα, and C-reactive protein (CRP). In contrast, intracellular pathogens, such as viruses, elicit a distinct antiviral gene program that involves the induction of type I interferon (IFN) genes via transcription factors such as interferon regulatory factors (IRFs). This anti-viral gene expression program mediates fundamentally different effector responses, which are categorized in this analysis to include lymphocyte proliferation and response to vaccination. In addition to these two broad categories of effector responses, enumerative measures characterize the relative distribution of lymphocyte subpopulations such as CD4 T-cells and NK cells.

### Data Extraction and Quality Assessment

Data extraction and quality assessment were performed by one investigator and confirmed by at least one other investigator. Disagreements were resolved by consensus among team members. We extracted information on study characteristics, population characteristics, and type, duration, frequency of interventions and immune outcomes. We also assessed the risk of bias for each study using the Cochrane risk of bias tool [33], with an overall rating as high, moderate, or low risk of bias (**Table S2**).

### Synthesis and Analysis

We qualitatively synthesized all included studies in summary tables. Studies were grouped into the following three categories of immune outcomes, according to the role of the central nervous system in regulating immune response genes programs and the effects of sympathetic activation on these effector responses and enumerative measures [28]: (1) Inflammatory (CRP, IL-6, TNF-α, and IL-8); (2) Anti-viral (Interferon-γ, lymphocyte proliferation, viral antibodies, and natural killer (NK) cytotoxicity); and (3) Enumerative measures (number of CD4 lymphocytes, NK cells, and leukocytes) and other immune-related measures (e.g. complement, IgA). See **Table 2** for the classifications.

**Table 2:** Summary of Evidence Reviewed Categorized by Inflammatory and Antiviral Outcomes.

Outcome of Interest	Mind-body therapy study [Reference]	Intervention Duration (Weeks)	Total Sample Size Analyzed	Risk of Bias	Summary of Key Findings for the Effect of Mind-body Therapies Compared with Control Interventions
**1. Inflammatory measures**					
CRP	Chen 2010 (TC) [68], Creswell 2012 (Med) [37], Irwin 2012; Irwin 2007 (TC) [60], [61], Lavertsky 2011 (TC) [67], Malarkey 2013 (Med) [51], Oh 2012; Oh 2010; Oh 2008 (QG) [72]–[74], Oken 2010 (Med) [43], Pullen 2010 (Yoga) [55], Pullen 2008 (Yoga) [54]	7 to 16	710	4 Moderate; 5 Low	**Finding 1.** Our random-effects meta-analysis of 9 studies showed a medium effect on decreasing CRP (ES 0.58, 95% CI 0.04 to 1.12, P = 0.035). There was a statistically significant heterogeneity across studies (I^2^ = 97%, P<0.001). **Finding 2.** Stratified by clinical populations, the subgroup meta-analysis showed that studies in healthy people were homogenous and had an insignificant small effect on CRP (ES 0.2, 95% CI. −0.13 to 0.53), with an I^2^ = 0%, while studies in populations with disease conditions were heterogeneous and showed a significant effect on CRP (0.74, 95% CI. 0.00 to 1.48]), with an I^2^ = 94%
IL-6	Chen 2006 (QG) [69], Creswell 2012 (Med) [37], Irwin 2012; Irwin 2007 (TC) [60], [61], Janelsins 2011[63], Sprod 2012 (TC) [65], Malarkey 2013 (Med) [51], McCain 2008 (TC) [64], Oken 2010 (Med) [43], Pace 2009 (Med) [44], Pullen 2008 (Yoga) [54], Pullen 2010 (Yoga) [55], Zautra 2008 (Med) [49]	6 to16	594	2 High; 2 Moderate; 7 Low	**Finding 1.** Our random-effects meta-analysis of 10 studies showed a small effect on decreasing IL-6 (ES 0.35, 95% CI −0.04 to 0.75, P = 0.08). There was a statistically significant heterogeneity across studies (I^2^ = 83%, P<0.001). Stratified by clinical populations, the subgroup meta-analysis showed no significant difference in the pooled effect on IL-6 between studies in healthy people and those in the population with disease conditions (ES 0.35 [95% CI. −0.22, 0.92] vs. 0.38 [95% CI. −0.23, 0.99], P_between_ = 0.91). **Finding 2.** 1 study not in meta-analysis reported “no significant changes” in cytokine levels (data not reported) following 10 weeks of Tai Chi or wait-list control in HIV patients.
TNF-α	Elsenbruch 2005 (Med) [40], Manzaneque 2009 (QG) [71], McCain 2008 (TC) [64], Oken 2010 (Med) [43], Rao 2008 -2 articles (Yoga) [56], [59]	4 to 10	415	2 High; 1 Moderate; 2 Low	**Finding 1.** Our random-effects meta-analysis of 3 studies showed a negligible to small effect on TNF-α (ES 0.21, 95% CI −0.15 to 0.58, P = 0.25). There is no significant heterogeneity across studies (I^2^ = 0%, P = 0.75). **Finding 2.** 2 studies not in meta-analysis both reported no significant changes in TNF-α comparing mind-body therapies to control interventions.
IL-8	Janelsins 2011 [63], Sprod 2012 (TC) [65], Barrett 2012 (Med) [36], Rozenkranz 2013 (Med) [45]	8 to12	158	1 High; 2 Low	1 study showed significant increases in IL-8 from nasal wash during upper respiratory infection while 2 studies showed no significant change in IL-8 from serum or blister fluid.
**2. Anti-viral measures**					
IFN-γ	Gopal 2011 (Yoga) [53], Janelsins 2011 [63], Sprod 2012 (TC) [65], Manzaneque 2009 (QG) [71], McCain 2008 (TC) [64]	4 to 12	228	1 High; 1 Moderate; 2 Low	3 studies (two in healthy individuals and one in breast cancer survivors) found no significant differences in cytokines (including IFN- γ), while one study found a large significant effect on IFN- γ among participants with HIV.
Lymphocyte Proliferation and vaccination responses	Irwin 2003 (TC) [62], Irwin 2012; Irwin 2007 (TC) [60], [61], McCain 2008 (TC) [64], Davidson 2003 (Med) [39]	8 to 16	318	3 Low; 1 High	**Finding 1.** 1 study found no significant effect immediately post-intervention but the overall lymphocyte function significantly improved versus control group at 6-month follow-up in patients with HIV. **Finding 2.** 2 studies showed a significant effect on VZV-RCF at rest and in response to vaccination in healthy older adults. **Finding 3.** 1 study also found significant increases in influenza antibody titer following influenza vaccination in healthy adults.
NK Cytotoxicity	McCain 2008 (TC) [64]	10	252	1 Low	1 study showed no significant change in NK cytotoxicity.
**3. Enumerative measures**					
CD4 Lymphocytes	Cade 2010 (Yoga) [52], Creswell 2009 (Med) [38], Elsenbruch 2005 (Med) [40], Hidderley 2004 (Med) [42], Lengacher 2013 (Med) [50], Manzaneque 2004 (QG) [70], McCain 2008 (TC) [64], SeyedAlinaghi 2012 (Med) [46], Solberg 1995 (Med) [47], Taylor 1995 (Med) [48], Wang 2011 (TC) [66]	4 to 20	777	8 High; 1 Moderate; 2 Low	**Finding 1.** Our random-effects meta-analysis of 7 studies showed a negligible to small effect on the number of CD4+* T lymphocytes (ES 0.15, 95% CI −0.04 to 0.34, P = 0.13). There is no significant heterogeneity across studies (I^2^ = 0%, P = 0.79). **Finding 2.** 3 studies (1 in healthy individuals and 1 in HIV patients) reported no significant changes in the number of CD4*+ T lymphocytes, and 1 study in healthy college students reported a significant increased in CD4+ T lymphocyte count in Tai Chi but not in control group.
NK Cells	Elsenbruch 2005 (Med) [40], Hidderley 2004 (Med) [42], Lengacher 2013 (Med) [50]	6 to 8	143	3 High	Our random-effects meta-analysis of 3 studies showed a negligible to small effect on the number of NK cells ES 0.12, 95% CI −0.21 to 0.45, P = 0.47). There is no significant heterogeneity across studies (I^2^ = 0%, P = 0.48)
Salivary IgA	Fan 2010 (Med) [41], Vogler 2011 (Yoga) [58]	4 to 8	73	2 High	1 study reported a significant increase in salivary IgA compared to the control (P = 0.03), and another study reported no significant difference in salivary IgA between groups.
Total blood count	Subramanian 2012 (Yoga) [57]	6	40	1 High	1 study reported a decrease in neutrophils (P<0.01) and increase in lymphocytes (P<0.01) compared to the control group.

Due to disparate measures in variable metrics, we calculated a standardized mean difference (Cohen’s *d*) comparing mind-body therapy with control (variable across studies). **Table 3** summarizes the technical details on the interpretations for effect size (ES). We obtained missing or unclear data through various techniques (e.g., contacting authors, imputing missing standard deviations or digitizing data from the result figures). Detailed information on the techniques is available upon request. In view of significant heterogeneity, the DerSimonian-Laird random-effect model was used for pooling [34]. Heterogeneity was estimated with the Cochran Q statistic (considered significant when the P value was less than 0.10) and quantified the extent of heterogeneity with the I^2^ index [35]. To explain the heterogeneity across studies, we conducted subgroup meta-analyses stratified by 1) clinical populations (healthy or with diseases), 2) types of MBTs (Tai Chi/Qi Gong, Yoga, or meditation), and 3) types of controls (defined by the original studies). The difference between subgroups was tested using a z-test. All analyses were conducted by using Stata SE 12 software (Stata Corp., College Station, Texas).

**Table 3:** Technical details on the interpretations for effect size (ES).

**Direction of the ES**	For consistency in the direction of ES across immune parameters, we calibrated the effect sizes for inflammatory markers with a negative orientation, such that an increase in effect corresponded to a reduction in inflammatory marker comparing mind-body therapy with control, by multiplying the mean change from baseline by (−1).
**Magnitude of the ES**	The magnitude of the ES (clinical effects) indicates: 0–0.19 = negligible effect, 0.20–0.49 = small effect, 0.50–0.79 = moderate effect, and 0.80(+) = large effect [103], [104].

## Results

**Figure 1:**
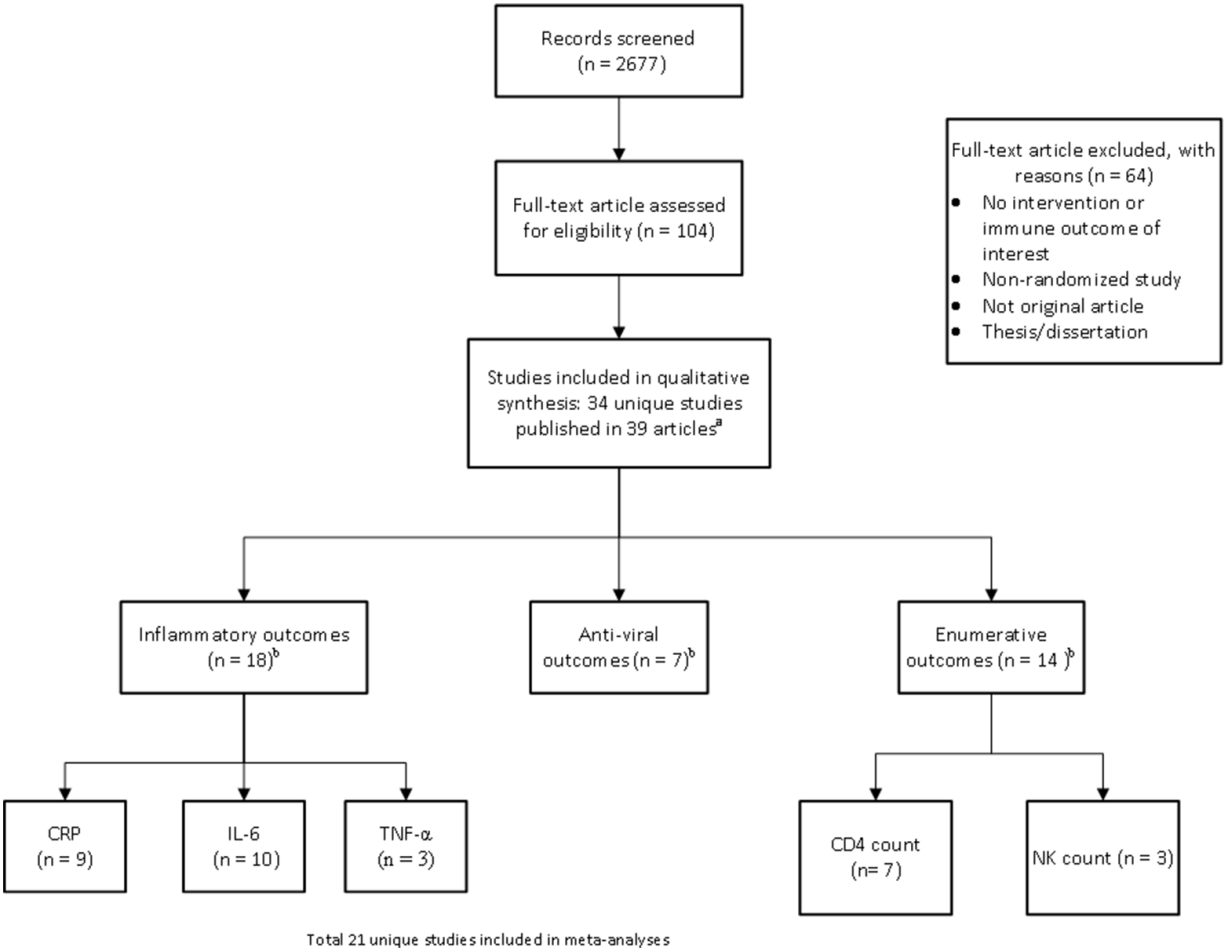
Literature Search and Study Selection. Legends: CD4 = cluster of differentiation 4 protein; CRP = c-reactive protein; IL-6 = interleukin-6; INF-γ = Interferon-gamma; NK count = natural killer cell count; TNF-α = Tumor necrosis factor. ^a^The studies were conducted in 9 countries (United States, China, India, Australia, Spain, Germany, Iran, Norway, and the United Kingdom). ^b^The sum of study number exceeds the total number of studies included due to some studies reported multiple outcomes across categories.

**Figure 1** depicts the literature search and study selection process. A total of 34 unique studies within 39 articles published from 1995–2013 were ultimately included.

**Table 2** summarizes the evidence reviewed according to types of outcomes. Of the 34 studies (published in 39 articles) [36]–[74], 16 of them evaluated meditation as the primary intervention [36]–[51], 7 evaluated Yoga [52]–[59], 7 evaluated Tai Chi [60]–[68], and 4 evaluated Qi Gong [69]–[74]. There were 984 healthy individuals (n = 17), and 1365 patients with chronic conditions such as: cancer (n = 5), HIV (n = 5), cardiovascular risk or disease (n = 3), depression (n = 1), diabetes (n = 1), rheumatoid arthritis (n = 1), and ulcerative colitis (n = 1). Mean age ranged from 21 to 70 years. Frequency and duration of interventions ranged from one to seven times/week for four to twenty weeks. Controls were varied, including wait-list, routine activity, and standard medical care. **Table 4** describes the characteristics of the 34 included studies.

**Table 4:** Study characteristics of the 34 randomized controlled trials evaluating the effects of mind-body therapies on the immune system.

Study [Reference]	Population	N Analyzed (% Female)*	Mean age (range), year	Intervention Duration, Type	Intervention Frequency, Style	Control Condition	Risk of Bias
Barrett 2012, USA [36]	Healthy adults >50 years of age reporting at least two colds in the past 12 months or average of 1 or more colds per year	102 (82%)	59	8 weeks; *Meditation*	2.5 hrs, 1 time(s)/week including individual practice recommended 45 min/wk; *MBSR*	Observational control	Low
							
Cade 2010, USA [52]	Adults diagnosed with HIV with CD4>200 and at least one cardiovascular disease risk factor	50 (26%)	45(18–70)	20 weeks; *Yoga*	60 min, 2–3 time(s)/week including individual practice recommended 1 time(s)/week; *Ashtanga Vinyasa*	Standard of care	Moderate
Chen 2006, Taiwan [69]	Healthy middle-aged women	87 (100%)	45	12 weeks; *Qi Gong*	3 time(s)/week; *Baduanjin, 8 sections*	Daily activities	High
Chen 2010, Taiwan [68]	Adults with diagnosis of Type II diabetes and BMI 30–35	104 (43%)	58	12 weeks; *Tai Chi*	60 min, 3 time(s)/week; *Chen-Style Tai Chi Chuan 99-form*	Conventional aerobic exercise	Moderate
Creswell 2009, USA [38]	Adults diagnosed with HIV and with at least minimal psychological distress	48 (10%)	41	8 weeks; *Meditation*	2 hrs, 1 time(s)/week including 1 day-long retreat and individual practice recommended daily; *MBSR*	6 hrs, 1 day; *Condensed MBSR stress-reduction education seminar*	Low
Creswell 2012, USA [37]	Healthy older adults	40 (80%)	65 (55–85)	8 weeks; *Meditation*	2 hrs, 1 time(s)/week including individual practice recommended 30 min/day; *MBSR*	Wait-list control	Low
Davidson 2003, USA [39]	Healthy adults	41 (71%)	36 (23–56)	8 weeks; *Meditation*	2.5–3 hrs, 1 time(s)/week including 1 day retreat and individual practice recommended 60 min, 6 time(s)/week; *MBSR*	Wait-list control	High
Elsenbruch 2005, Germany [40]	Adults diagnosed with ulcerative colitis	30 (67%)	42	10 weeks; *Meditation*	6 hrs, 1 time(s)/week; *Mind-Body intervention program based on the mind-body and MBSR programs*	Wait-list Control	High
							
Fan 2010, China [41]	Healthy college students	35 (51%)	21	4 weeks; *Meditation*	20 min, 7 time(s)/week; *Integrative body- mind training (IBMT) that consists of body relaxation, breathing \adjustment, mental imagery, and mindfulness training*	Muscle relaxation training	High
Gopal 2011, India [53]	Healthy female students	60 (100%)	17–20	12 weeks; *Yoga*	35 min, 7 time(s)/week; *Integrated Yoga*	Normal routine	High
							
Hidderley 2004, UK [42]	Women with early stage breast cancer post-lumpectomy and adjuvant radiation	31 (100%)	16–65	8 weeks; *Meditation*	1 time(s)/week; *Autogenic training plus home visit*	Home visit alone	High
Irwin 2007 [60]; Irwin 2011 and 2012, USA [60], [61]	Healthy older adults with history of varicella confirmed by VZV-CMI response,	112 (63%)	70 (59–86)	16 weeks; *Tai Chi*	40 min, 3 time(s)/week for a total of 120 minutes *Tai Chi Chih with 20 exercises*	Health education consisting of 16 didactic presentations on a series of health related themes	Low
Irwin 2003, USA [62]	Healthy older adults with history of varicella or long-term (over 30 years) residence in the continental US	36 (72%)	71	15 weeks; *Tai Chi*	45 min, 3 time(s)/week for a total of 45 sessions; *Tai Chi Chih with 20 standardized movements*	Wait-list control (maintenance of routine activities)	Low
Janelsins, 2011 [63]; Sprod 2012, USA [65]	Breast cancer survivors who recently completed treatment	19 (100%)	53 (43–78)	12 weeks; *Tai Chi*	60 min, 3 time(s)/week; *Yang style Tai Chi Chuan, 15-move short form*	Standard support consisting of psychosocial therapy	Low
Lavretsky 2011, USA [67]	Elderly adults with major depression	73 (62%)	71	10 weeks; *Tai Chi*	120 min, 1 time(s)/week	Health education	Low
Lengacher, 2013, USA [50]	Women with stage 0–3 breast cancer post lumpectomy and adjuvant radiation +/− chemotherapy,	82 (100%)	58	6 weeks; *Meditation*	2 hr, 1 time(s)/week; *MBSR adapted for breast cancer*	Usual care (standard post-treatment clinic visits)	High
Malarkey 2013, USA [51]	Adults with elevated CRP >3.0 mg/ml and at risk for or with known cardiovascular disease	186 (88%)	50	8 weeks; *Meditation*	1 hr, 1 time(s)/week including individual practice recommended 20 min/day; *Mindfulness Based intervention adapted from MBSR*	Lifestyle Education	Low
Manzaneque 2004, Spain [70]	Healthy college students	29 (52%)	18–21	4 weeks; *Qi Gong*	30 min, 7 time(s)/week group + variable individual practice; *Ba Duan Jin, 8 movements repeated 8 times for a total of 64 movements*	Daily activities	High
Manzaneque 2009, Spain [71]	Healthy college students	33 (88%)	18–21	4 weeks; *Qi Gong*	30 min, 3 time(s)/week group + variable individual practice; *Ba Duan Jin, 8 movements repeated 8 times for a total of 64 movements*	Daily activities	Moderate
							
McCain 2008, USA [64]	Adults with diagnosis of HIV	125 (40%)	42	10 weeks; *Tai Chi*	90 min, 1 time(s)/week; *Focused short form Tai Chi training with 8 movements*	Wait-list control	Low
Oh 2008, 2010, and 2012, Australia [72]–[74]	Cancer patients who had received chemotherapy or were undergoing chemotherapy	162 (57%)	60 (31–86)	10 weeks; *Qi Gong*	90 min, 2 time(s)/week + individual practice recommended 30 minutes per day; *Medical Qi Gong*	Usual Care	Moderate
Oken 2010, USA [43]	Healthy adults caring for a family member with dementia and with high baseline stress	19 (76%)	65 (45–85)	7 weeks; *Meditation*	90 min, 1×per week; *Adapted from MBSR and CBT*	Education clas	Low
Pace 2009, USA [44]	Healthy college students	61 (52%)	19 (17–19)	6 weeks; *Meditation*	50 min, 2 time(s)/week including individual practice; *Compassion meditation derived from Tibetan lojong practice*	Health discussion group	High
Pullen 2008, USA [54]	Adults with heart failure (NYHA Class I-III)	19 (53%)	51	8 weeks; *Yoga*	70 min, 2 time(s)/week including individual practice recommended >3 time(s)/week; *Hatha Yoga plus standard medical therapy*	Standard medical therapy	Moderate
Pullen 2010, USA [55]	African American adults with heart failure (NYHA Class I-III)	40 (43%)	54 (31–76)	8–10 weeks; *Yoga*	60 min, 2 time(s)/week; *Hatha Yoga*	Standard medical therapy	Moderate
Rao 2008, India [56], [59]	Women with stage II-IV breast cancer undergoing surgery	69 (100%)	49	4 weeks; *Yoga*	4 sessions in hospital followed by individual practice guided by audiotape; *Integrated Yoga program*	Supportive counseling and postoperative exercise rehabilitation	High
							
Rosenkranz 2013, USA [45]	Healthy adults	49 (80%)	46 (19–59)	8 weeks; *Meditation*	2.5 hrs, 1 time(s)/week including individual practice recommended 45–60 min/day; *MBSR*	Health Enhancement Program	High
SeyedAlinaghi 2012, Iran [46]	Adults diagnosed with HIV with CD4 count >250 not on antiretroviral therapy	173 (31%)	35.1	8 weeks; *Meditation*	Not reported; *MBSR*	Education and Support	High
Solberg 1995, Norway [47]	Men regularly engaged in exercise	12 (0%)	43 (27–49)	7 weeks; *Meditation*	30 min sequences of regular home practice*; Meditation consisting of single sound repetition*	No meditation or formal relaxation	High
Subramanian 2012, India [57]	Healthy college students (both female and male)	40 (not reported)	18–23	6 weeks; *Yoga*	6 day course followed by individual practice recommended daily; *Sudarshan Kriya and Pranayam*	Unspecified	High
							
Taylor 1995, USA [48]	Men diagnosed with HIV, asymptomatic with CD4 T lymphocyte counts <400	10 (0%)	28–44	10 weeks; *Meditation*	1 hr, 2 time(s)/week; *Behavioral stress-management consisting of progressive muscle relaxation, EMG biofeedback, self-hypnosis and secular meditation*	No treatment	High
							
Vogler 2011, Australia [58]	Physically inactive elderly adults	38 (not reported)	73 (56–94)	8 weeks*; Yoga*	90 min, 2 time(s)/week including individual practice recommended 20 min, 3 time(s)/week; *Iyengar Yoga*	Wait-list control (daily routine)	High
Wang 2011, China [66]	Healthy sedentary female college students	60 (100%)	19	12 weeks*; Tai Chi*	45 min, 5 time(s)/week; *Tai Chi Chuan, 24 standardized movements*	Routine activity	High
							
Zautra 2008, USA [49]	Adults diagnosed with rheumatoid arthritis	144 (68%)	56	8 weeks; *Meditation*	2 hrs, 1 time(s)/week; *Mindfulness meditation and emotion regulation therapy modified from MBSR (excluding Yoga and day-long retreat)*	Education-only	Low
							
							

CD4 = cluster of differentiation 4 protein; CRP = c-reactive protein; IL-6 = interleukin 6; IL-8 = interleukin 8; INF-gamma = Interferon gamma; Med = meditation; NK count = natural killer cell count; QG = Qi Gong; TC = Tai Chi; TNF-α = Tumor necrosis factor.

min = minutes; hr = hour; hrs = hours.

*In studies that involved more than one active intervention, we restricted our analyses to the comparisons between mind-body therapy intervention and control group. Therefore, number analyzed reflects the total number of participants analyzed in the mind-body therapy intervention and control group only.

Twenty one of the 34 studies were meta-analyzed for one or more outcomes (**Figure 1**). We describe below results for each outcome separately for studies that provided data for meta-analysis and those excluded from meta-analysis.

### 1. Inflammatory Outcomes

Eighteen studies published in 22 articles [11], [36], [37], [40], [43], [45], with a total of 1,667 participants reported data on the effects of MBTs on inflammatory outcomes (i.e. CRP, IL-6, TNF-α, and IL-8). Of these 18 studies, four were rated at high risk, five at moderate risk, and nine at low risk of bias.

**a. CRP.** Nine studies [37], [43], [51], [54], [55], [60], [61], [67], [68], [72]–[74] evaluated the effects of MBTs [meditation (n = 3), Tai Chi (n = 3), Qi Gong (n = 1), and Yoga (n = 2)] on CRP in 710 participants with a variety of clinical conditions (heart failure [54], [55], Type 2 diabetes [68], major depression [67], healthy individuals [37], [43], [60], [61], elderly participants with cardiovascular disease risk factors [51], or cancer patients [72]–[74]). Various controls were compared among the nine trials including, wait-list (n = 1); aerobic activity (n = 1); education (n = 4); and usual care (n = 3).

**Figure 2:**
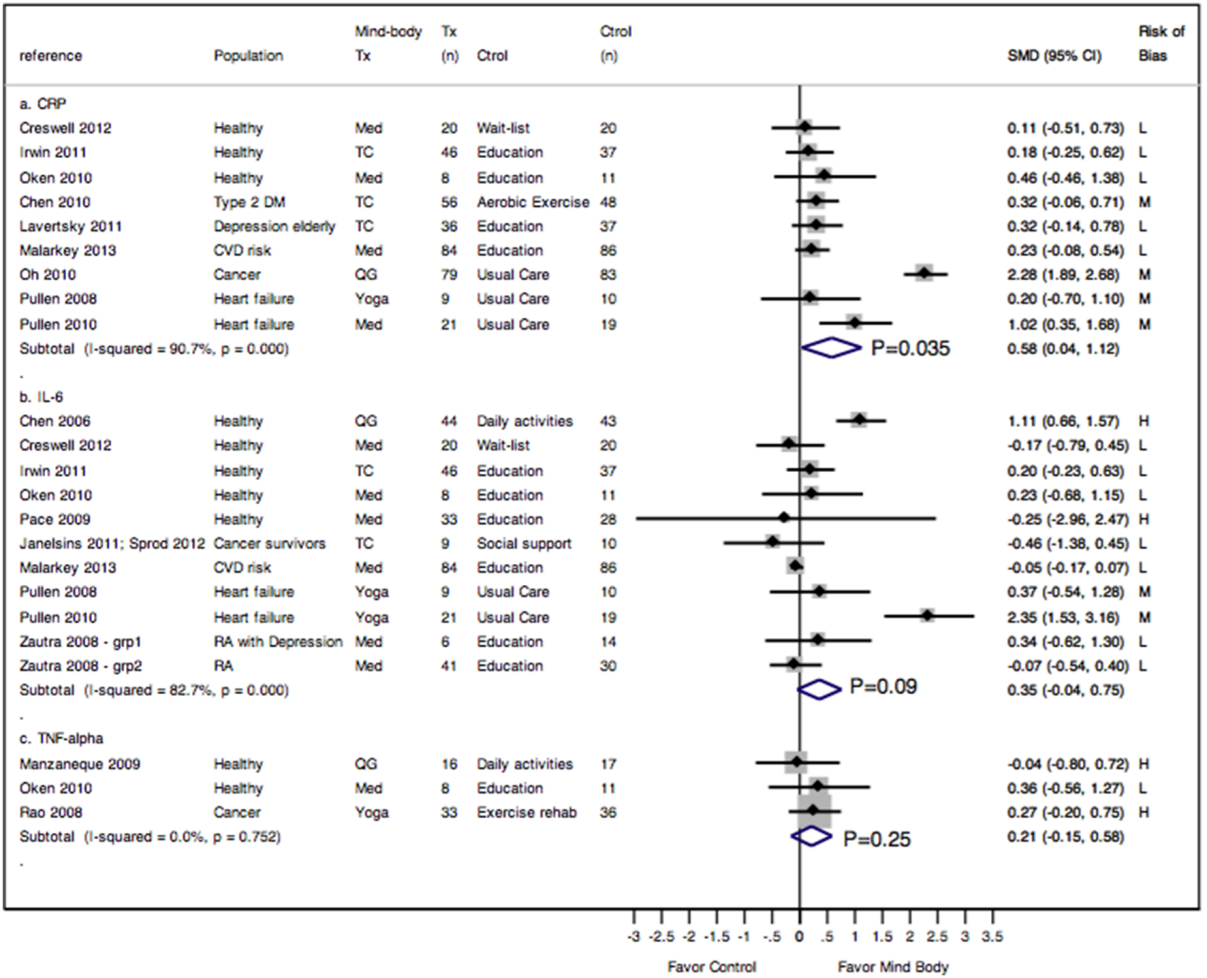
Meta-analysis of RCTs comparing the effect of mind-body therapies with control interventions on inflammatory markers: CRP (panel a), IL-6 (panel b), and TNF-α (panel c). Legends: CRP = c-reactive protein; IL-6 = interleukin 6; Med = meditation; QG = Qi Gong; TC = Tai Chi; TNF-α = Tumor necrosis factor; Tx = treatment; RA = rheumatoid arthritis; SMD = standardized mean difference. Risk of bias: L = low; M = medium; H = high (see Table S2 for details). Zautra 2008 reported only subgroup results (grp 1 = RA patients with depression; grp 2 = RA patients without depression) and data from each subgroup were entered in the meta-analysis separately. P-values adjacent to I-squared results are p-values for heterogeneity testing (P<0.05 indicates significant heterogeneity), and p-values adjacent to meta-analysis pooled results (diamonds) are p-values for the pooled effect sizes.

**Figure 3:**
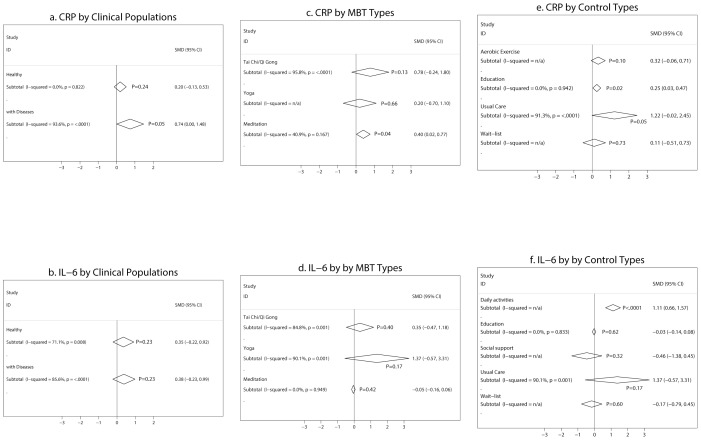
Subgroup meta-analysis of RCTs comparing the effect of mind-body therapies with control interventions on CRP and IL-6 by clinical populations (panels a and b), by MBT types (panels c and d), and by control types (panels e and f). Legends: SMD = standardized mean difference. MBT = mind-body therapies. P-values adjacent to I-squared results are p-values for heterogeneity testing (P<0.05 indicates significant heterogeneity and “I-squared = n/a” indicates that there was only 1 study in the subgroup so heterogeneity was not applicable), and p-values adjacent to meta-analysis pooled results (diamonds) are p-values for the pooled effect sizes.

Our meta-analysis showed that 7 to 16 weeks (1 to 3 times/week totaling 60 to 180 minutes weekly) of MBTs demonstrated medium effect and statistically significant improvements on CRP compared with control interventions (ES 0.58, 95% CI. 0.04 to 1.12, P = 0.04) (**Figure 2** **, panel a**), with an I^2^ = 97%. Stratified by clinical populations, the subgroup meta-analysis showed that studies in healthy individuals were homogeneous and showed an insignificant effect on CRP (ES 0.2, 95% CI. −0.13 to 0.53), with an I^2^ = 0%. On the contrary, studies in populations with disease conditions were heterogeneous and showed a significant effect on CRP (ES 0.74, 95% CI. 0.00 to 1.48), with an I^2^ = 94% (**Figure 3** **, panel a**). However, the difference in the pooled effect sizes between the two subgroups was not significant (P_between_ = 0.34).

Further subgroup analysis exploring the effects of different MBTs on CRP showed that studies of Tai Chi/Qi Gong had larger but more heterogeneous mean effect sizes (ES 0.78, 95% CI. –0.24 to 1.80; I^2^ = 96%) compared with studies of meditation (ES 0.40, 95% CI. 0.02 to 0.77; I^2^ = 41%). The single study that investigated Yoga found an insignificant effect on CRP (ES 0.20, 95% CI. –0.70 to 1.10) (**Figure 3** **, panel c**).

We also performed a subgroup meta-analysis on CRP by types of controls. The results showed that the effect sizes ranged widely across different types of controls (**Figure 3** **, panel e**)**.**

**b.** **IL-6.** Eleven studies [37], [43], [44], [49], [51], [54], [55], [60], [61], [63]–[65], [69] evaluated the effects of 6 to 16 weeks of MBTs [meditation (n = 5), Tai Chi (n = 3), Yoga (n = 2), and Qi Gong (n = 1)] on IL-6. Ten studies with 565 participants were included in our meta-analysis [37], [43], [44], [49], [51], [54], [55], [60], [61], [63], [65], [69], but one study with 125 participants lacked sufficient quantitative detail for meta-analysis [64]. Among the eleven trials, various control conditions included: wait-list (n = 1); aerobic activity (n = 1) and social support (n = 1); education (n = 6); and usual care (n = 2).

Our meta-analysis of ten studies showed that 6 to 16 weeks (1 to 3 times/week totaling 60 to 180 minutes of weekly instructions) of MBTs demonstrated a small, but not statistically significant effect on IL-6 (0.35, 95% CI - 0.04 to 0.75, P = 0.08) compared with control interventions (**Figure 2** **panel b**), with an I^2^ = 83%. Stratified by clinical populations, the subgroup meta-analysis showed no significant difference in the pooled effect on IL-6 between studies in healthy individuals and those with disease conditions (ES 0.35 [95% CI. −0.22, 0.92] vs. 0.38 [95% CI. −0.23, 0.99], P_between_ = 0.91) (**Figure 3** **, panel b**).

Similar to CRP, studies of Tai Chi/Qi Gong (ES 0.35, 95% CI. −0.47 to 1.18; I^2^ = 84%) and Yoga (ES 1.37, 95% CI. −0.57 to 3.31; I^2^ = 90%) had larger but more heterogeneous mean effect sizes on IL-6 compared with that of meditation (ES −0.05, 95% CI. −0.16 to 0.06; I^2^ = 0%) (**Figure 3** **, panel d**). The meta-analysis also showed that the effect sizes on IL-6 ranged widely across different types of controls (**Figure 3** **panel f**).

One study in HIV patients’ self- reported “no significant changes” in cytokine levels (no data in meta-analysis) following 10 weeks of Tai Chi or wait-list control [64]. Overall, 11 studies suggest that MBTs were associated with small but not significant effects on IL-6 compared with control interventions.

**c. TNF-α.** Five studies [40], [43], [56], [59], [64], [71] evaluated the effects of 4 to 10 weeks of MBTs [meditation (n = 2), Qi Gong (n = 1), and Yoga (n = 1)] on TNF-α. Three studies with 121 participants were included in our meta-analysis [40], [43], [56], [59], [64], [71], while two studies with 84 participants lacked sufficient quantitative detail and were not included in the analysis [40], [43], [56], [59], [64], [71].

Our meta-analysis of three studies showed that 4 to 7 weeks (1 to 7 times/week totaling 90 to 210 minutes weekly) of MBTs showed a negligible to small effect on TNF-α (ES 0.21, 95% CI. –0.15 to 0.58, P = 0.25) (**Figure 2** **, panel c**), with an I^2^ = 0%. The two studies which were not included in the meta-analysis reported no significant differences in TNF-α after 10 weeks of meditation [40] or Tai Chi [64]. Together, these five studies suggest that MBTs do not have a significant effect on TNF-α, compared with control interventions.

**d. IL-8.** For IL-8, three studies [36], [45], [63], [65] with a total of 158 participants were identified. Meta-analysis could not be performed due to insufficient data. One study of 149 healthy adults, rated at low risk of bias, found that eight weeks of meditation (2.5 hours, 1 time/week) led to significantly higher levels of IL-8 in nasal wash collected during acute respiratory infection compared to control (P = 0.02) [36]. The other two (one rated at low risk [63] and one at high risk [45]) reported that 10 and 12 weeks of Tai Chi or meditation did not show significant changes in IL-8 compared with control interventions.

Overall, MBTs were positively associated with decreased CRP levels in patients with type 2 diabetes, cancer, or heart failure, and the elderly with depression and cardiovascular disease risk factors. Small but insignificant improvements were also noted in levels of IL-6. The impact of mind-body therapies on TNF-α and IL-8 remains uncertain.

### 2. Anti-Viral Related Outcomes

A total of seven studies published in nine articles [39], [53], [60]–[65], [71] reported data on the effects of MBTs on anti-viral outcomes such as IFN-γ (n = 4), lymphocyte proliferation including viral-specific, cell-mediated immune responses (i.e., varicella zoster virus responder cell frequency) [VZV-RCF] (n = 3), viral antibodies (n = 1), and NK cytotoxicity (n = 1). Studies evaluated 4 to 16 weeks of meditation (n = 1), Tai Chi (n = 4), Yoga (n = 3), and Qi Gong (n = 3). Due to the paucity of studies and heterogeneity of outcomes, meta-analysis could not be performed.

For IFN-γ, four studies [Tai Chi (n = 2), Qi Gong (n = 1), and Yoga (n = 1)] with 228 participants and a variety of clinical conditions (healthy individuals, HIV positive patients, and breast cancer survivors) were identified [53], [63]–[65], [71]. Three studies (two with healthy individuals and one with breast cancer survivors) found no significant differences in cytokines (including IFN-γ) comparing mind-body therapy with controls [53], [63], [65], [71]. On the contrary, one study which compared Tai Chi (90 minutes, 1 time per week) with wait-list control found a large significant effect on IFN-γ among 119 patients with HIV. The study was rated low risk of bias [64].

For lymphocyte proliferation and vaccination responses**,** four studies [three Tai Chi (all rated at low risk of bias) and one meditation (rated at high risk of bias)] were identified [39], [60]–[62], [64]. One study evaluated the effect of Tai Chi on lymphocyte function (measured by lymphocyte proliferation assays) in patients with HIV [64]. The overall lymphocyte function significantly improved as compared to the control group at six-month follow-up. Two other studies compared effects of Tai Chi versus health education on VZV-RCF in 148 healthy older adults, and both found a significant effect on VZV-RCF at rest [60], [62] and in response to vaccination [61]. Finally, one other study also examined anti-viral antibodies following influenza vaccination by comparing an eight-week meditation with the wait-list control among 41 healthy adults [39]. Although rated at high risk of bias, this study reported significantly greater increases in anti-influenza antibody titers at the four week and eight-week follow-up compared with the control.

For NK cytotoxicity, only one study of 119 HIV patients [64], rated at low risk of bias, reported that Tai Chi (90 minutes, 1 time per week) had an insignificant effect on NK cytotoxicity compared with wait-list control.

### 3. Other enumerative Outcomes

A total of 14 studies [38], [40]–[42], [46]–[48], [50], [52], [57], [58], [64], [66], [70] reported data on the effects of MBTs on enumerative outcomes, such as CD4 cells (n = 11), NK cells (n = 4), salivary IgA (n = 2), and total blood count (n = 1). Of these 14 studies, 11 were rated at high risk, one at moderate risk, and two at low risk of bias.

**Figure 4:**
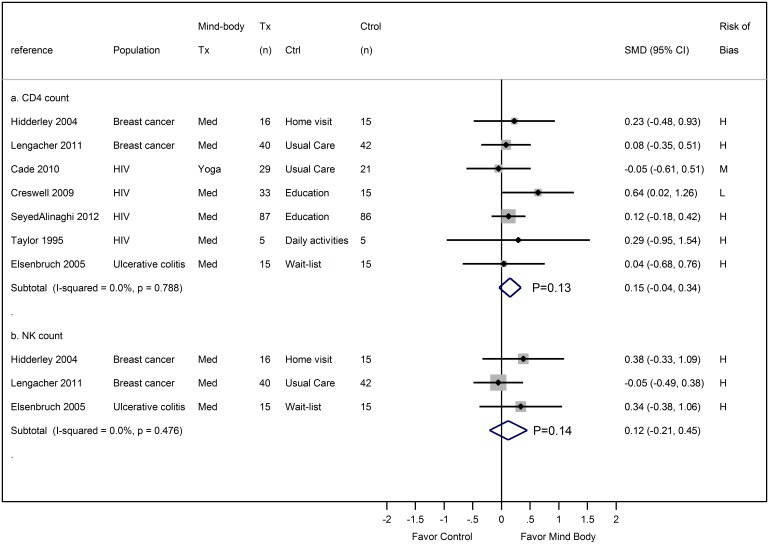
Meta-analysis of RCTs comparing the effect of mind-body therapies with control interventions on enumerative markers: CD4 count (panel a) and NK count (panel b). Legends: CD4 = cluster of differentiation 4 protein; Med = meditation; NK count = natural killer cell count; Tx = treatment; SMD = standardized mean.

Eleven studies [38], [40], [42], [46]–[48], [50], [52], [64], [66], [70] evaluated the effects of MBTs on CD4 in a total of 676 participants with diseases (HIV, breast cancer, and ulcerative colitis) and 101 healthy individuals. Our meta-analysis of seven studies with diseases showed that 6 to 10 weeks of meditation (1 to 2 times/week totaling 60 to 360 minutes weekly) and a 20-week Yoga practice (60 minutes, 2 to 3 times/week) had a negligible to small effect on CD4 count (0.15, 95% CI −0.04 to 0.34, P = 0.13) (**Figure 4** **panel a**), with an I^2^ = 0%. Four studies lacked sufficient quantitative data and were not included in the meta-analysis. Of these, two reported insignificant changes in CD4 count after four weeks (30 minutes, 7 times/week) of Qi Gong [70] or seven weeks (30 minutes, home practice) of meditation [47], compared with no interventions. Another study in college students reported a significant increase in CD4 count in Tai Chi (45 minutes, 5 times/week) but not in the control after 12 weeks [66]. The fourth study of 252 adults with HIV found no significant differences in the number of lymphocytes (CD4, CD8, and NK cells) between Tai Chi (90 minutes, 1 time/week) and wait-list controls after 10 weeks [64].

Three studies [40], [42], [50] evaluated the effects of meditation on NK counts in 30 patients with ulcerative colitis and 113 with breast cancer. The durations of meditation ranged from six to eight weeks (1 time per week totaling 60 to 360 minutes weekly). Our meta-analysis showed that meditation had a negligible to small effect on the number of NK cells (ES 0.12, 95% CI −0.21 to 0.45, P = 0.47) (**Figure 4** **panel b**), with an I^2^ = 0%.

For other outcomes, three studies with 113 participants reported data on the effects of MBTs [meditation (n = 1); Yoga (n = 2)] on salivary IgA and total blood count [41], [57], [58]. Results from these three studies were mixed (**Table 2**). All were rated at high risk of bias; thus no overall conclusion can be drawn.

## Discussion

This study extends our previous investigations of the effects of Tai Chi on health outcomes [3], [18]–[22], highlighting the promising role that MBTs may play in regulating the immune system. Overall, our findings suggest that MBTs may reduce inflammation, particularly among clinical populations, as evidenced by the significant reductions in CRP. In addition, a few high quality studies suggest that MBTs may increase virus-specific, cell-mediated immunity at rest and in response to vaccinations. Thus, the anti-inflammatory effects of MBTs, albeit incomplete, provide insight into the potential mechanisms behind mind-body treatment and the numerous health benefits they confer

Indeed, evidence accrued from 34 RCTs indicates that Tai Chi, Qi Gong, meditation, and Yoga, both short- and long-term, appear to reduce markers of inflammation and influence virus-specific immune responses to vaccinations, despite minimal evidence suggesting effects on resting anti-viral immunity or enumerative measures among 2219 healthy individuals and those with disease conditions. Specifically, for inflammatory measures, 18 published RCTs reported that 7 to 16 weeks of mind-body interventions significantly reduced C-reactive protein, and produced a small but nonsignificant reduction of IL-6, as well as a negligible effect on TNF-α. For anti-viral related immune and enumerative measures, among 21 trials there were negligible effects on CD4 count and natural killer cell counts with some evidence that mind-body interventions increase immune responses to vaccination. Our exploratory subgroup meta-analyses suggest that the possible contribution of Tai Chi or Qi Gong exercise to the immune outcomes, as its pooled effect size was larger than that of meditation. However, these differences in effect size may in part be explained by the heterogeneity in control conditions or populations studied.

Our findings are supported by existing literature evaluating the immunomodulatory effects of other types of behavioral interventions including exercise, stress reduction, and mood modifying approaches. For example, exercise, one of the most widely-studied behavioral interventions, has been shown to reduce chronic inflammation, enhance immunological memory in the context of vaccination, and even reduce sick days associated with the common cold and other upper respiratory tract infections [17], [29], [75]. Furthermore, we compared our meta-analysis results of the MBTs on CRP and IL-6 to other recognized interventions such as weight loss and lifestyle changes on vascular inflammatory markers in obese women (Esposito et al.) [76]. To perform this comparison, we converted data from Esposito et al. into standardized effect sizes (Cohen’s d). The results suggested that MBTs have a larger effect size (pooled effect size = 0.58; P = 0.035) than weight loss (effect size = 0.18; P = 0.008) on CRP, while the benefits are comparable on IL-6 (pooled effect size = 0.35; P = 0.09) versus weight loss (effect size = 0.349; P = 0.009) [76].

Major life adversities and psychological stress such as depression have divergent effects on the immune system; anti-viral immunity and resistance to infectious disease are reduced, whereas markers of inflammation and risk of inflammation associated disease are increased [28], [32], [75], [77], [78]. Initial efforts to understand this pattern of infectious- vs. inflammation- associated disease were unsuccessful by their focus on HPA axis activation and glucocorticoid-mediated suppression of an immune response in which both anti-viral and inflammatory responses are reduced [28]. In contrast, the sympathetic nervous system (SNS) has been found to inhibit anti-viral genes and to activate pro-inflammatory genes. For example, β-adrenergic signaling reduces anti-viral or adaptive immune responses by suppressing Th1-type gene expression (such as *IFNG* and *IL-12B*) and by stimulating transcription of T helper 2 (Th2)-type cytokine genes (such as *IL-4* and *IL-5*) [79]–[81]. Adrenergic signaling also leads to up-regulated transcription of pro-inflammatory cytokines such as *IL-1B*, *TNF* and *IL-6* [82], [83], consistent with evidence that acute psychological stress increases circulating levels of IL-6 and IL-1β [84]. However, other studies have found that adrenergic signaling can increase, as well as inhibit the production of IL-6 and TNF [85]–[89]. Nevertheless, adrenergic activation increases NF-κB in peripheral blood mononuclear cells (PBMCs) [84], [90], [91], and primes increases in the *ex vivo* production of pro-inflammatory cytokines in response to stimulation to lipopolysaccharide (LPS) and other Toll-like receptor ligands [90], [92]–[95]. Finally, social stressors that act in the long-term also lead to increased expression of pro-inflammatory immune response genes despite the presence of stable or elevated glucocorticoid levels [32], [75], [96]–[98]. To the extent that mind-body interventions are able to reverse the effects of acute and chronic stress and reduce SNS activation [99], a reversal of the anti-viral and inflammatory transcriptional bias of stress on immune response genes has been found [37], [100]–[102]. Indeed, both Tai Chi and mindfulness based meditation can reduce pro-inflammatory response gene profiles [37], [102], and a Yogic meditation appears to reverse increased NF-kB-related transcription of pro-inflammatory cytokines and decreased IRF1-related transcription of innate antiviral response genes [101].

Apparently, powerful links exist between the brain and the immune system, and psychosocial factors can directly influence health through behavior. MBTs may buffer these immune alterations through relaxation, stress reduction, improved mood, and moderate physical activity. Behavioral responses are therefore the key to activating neuroendocrine and autonomic pathways, which in turn modulate the immune system and have implications for susceptibility to a variety of diseases. These changes foreshadow a synergistic effect on down-regulating the sympathetic nervous system, causing diminished pro-inflammatory gene response [17], [29]–[31], [84]. Thus, behavioral interventions that alter immune responses provide potent evidence for psychological influences on immune function.

Our study is limited in a few important ways. *First*, studies were heterogeneous with regards to population characteristics, particularly with regard to age and health status, which limit the generalizability of our findings. Furthermore, it is possible that only populations under stress psychologically, physiologically, or with an inflammatory condition, will reveal changes in the immune markers measured in response to MBTs. *Second,* interventions were heterogeneous with regard to type, frequency, and duration. Moreover, the mind-body therapies may not have been administered at a sufficient dose or for sufficient duration to affect immune changes. In addition, we are unable to make any determination as to whether immunological outcomes resulted from a single component of the multi-dimensional intervention or rather from the intervention as a whole. *Third,* the outcome itself may not be sensitive to change with MBTs. For example, CRP is an acute phase protein that is induced by IL-6, and increases of CRP occur only following robust activation of IL-6.

Despite limitations, further methodologically rigorous evaluation of the effects MBTs on the immune system are warranted with emphasis on clinically applicable outcomes. In the future, mind-body studies should focus on elderly and clinical populations with identified immune deregulation to better elucidate potential implications for clinical outcomes. For example, such mind-body studies should test the effects of MBTs among populations with pro-inflammatory states such as infection, neoplasm, inflammatory arthritis, infarction, and tissue injury. Moreover, understanding dose response relationship and critical components of mind-body interventions associated with changes in hormone levels, inflammatory outcomes, and antiviral outcomes will be beneficial to understanding and unraveling mechanistic pathways

In summary, MBTs impact inflammatory measures and may influence virus-specific, cell-mediated immune responses to vaccination; there is minimal evidence to suggest effects on other related enumerative measures. Further understanding of the effects of MBTs on the immune system and clinical outcomes will provide insight into and affirm the phenomenon of complementary and alternative medicine therapeutic concepts, thus establishing a new paradigm for understanding health and treating illness.

## Supporting Information

Table S1 **Search strategy.**
(DOCX)

Table S2 Assessment of overall risk of bias*.
(DOCX)

Checklist S1 **PRISMA Checklist.**
(DOC)
