# Supplementary material for: The Effects of Mind-Body Therapies on the Immune System: Meta-Analysis
Source: PLoS One. 2014 Jul 2;9(7):e100903. doi: 10.1371/journal.pone.0100903 (PMC4079606; doi:10.1371/journal.pone.0100903)
Supplement: Table S1 — Search strategy. (DOCX) [file pone.0100903.s001.docx]

**Table S1. Search strategy**

1. **OVID MEDLINE**

| **Row** | **Intervention Variable** | **Search terms:** | **# of hits:** |
| --- | --- | --- | --- |
| 1 | Mind-Body Therapies | Mind-Body Therapies (MeSH) | 38878 |
| 2 |  | Mind-Body.mp | 2821 |
| 3 |  | Mind Body.mp | 2821 |
| 4 |  | OR 1-3 | 40517 |
| 5 | Tai Chi | Tai Ji (MeSH) | 498 |
| 6 |  | Tai Chi.mp | 613 |
| 7 |  | Tai Chi Chuan.mp | 105 |
| 8 |  | Tai Chi Chih.mp | 10 |
| 9 |  | T’ai Chi.mp | 613 |
| 10 |  | Tai ji.mp | 502 |
| 11 |  | Tai Ji Quan.mp | 2 |
| 12 |  | Taijiquan.mp | 8 |
| 13 |  | OR 5-12 | 675 |
| 14 | Qi Gong | Breathing Exercises (MeSH) | 2532 |
| 15 |  | Qi Gong.mp | 54 |
| 16 |  | Qigong.mp | 288 |
| 17 |  | Qi-gong.mp | 54 |
| 18 |  | Qi-training.mp | 37 |
| 19 |  | Chi Kung.mp | 5 |
| 20 |  | OR 14-19 | 2656 |
| 21 | Meditation | Meditation (MeSH) | 1250 |
| 22 |  | Meditat*.mp | 2768 |
| 23 |  | Mindful*.mp | 1840 |
| 24 |  | OR 21-23 | 4090 |
| 25 | Yoga | Yoga (MeSH) | 1308 |
| 26 |  | Yog*.mp | 4116 |
| 27 |  | OR 25-26 | 4116 |
| 28 | Combined Terms for Intervention Variable | 4 OR 13 OR 20 OR 24 OR 27 | 45339 |
| **Row** | **Outcome Variable** | **Search terms:** | **# of hits:** |
| 29 | Immune System | Immune System (MeSH) | 903214 |
| 30 |  | Immunoproteins (MeSH) | 758720 |
| 31 |  | Cytokines (MeSH) | 489586 |
| 32 |  | Immune System Phenomena (MeSH) | 631934 |
| 33 |  | Immun*.mp | 2035646 |
| 34 |  | Inflamm*.mp | 548960 |
| 35 |  | Leukocyte.mp | 123210 |
| 36 |  | Lymphocyte.mp | 246811 |
| 37 |  | Antibod*.mp | 892291 |
| 38 |  | Cytokine.mp | 124131 |
| 39 |  | C-reactive protein.mp | 38740 |
| 40 |  | OR 29-39 | 3357263 |
| 41 | Psychoneuroimmunology | Psychoneuroimmunology (MeSH) | 897 |
| 42 |  | Psychoneuroimmunology.mp | 1050 |
| 43 |  | OR 41-42 | 1050 |
| 44 | Combined Terms for Outcome Variable | 40 OR 43 | 3357466 |
| **45** | **Combined Terms for Intervention and Outcome** | **28 AND 44** | **1456** |
| **46** | **Limit to English Language** |  | **1289** |
| **47** | **Limit to Humans** |  | **1143** |

1. **PsychINFO**

| **Row** | **Intervention Variable** | **Search terms:** | **# of hits:** |
| --- | --- | --- | --- |
| 1 | Mind-Body Therapies | Su mind-body therapies  All mind-body  All mind body | 11230 |
| 2 | Tai Chi | Su Tai Ji  All Tai Chi  All Tai Chi Chuan  All Tai Chi Chih  All T’ai Chi  All Tai Ji  All Tai Ji Quan  All Taijiquan | 509 |
| 3 | Qi Gong | Su Breathing Exercises  All Qi Gong  All Qigong  All Qi-gong  All Qi-training  All Chi Kung | 525 |
| 4 | Meditation | Su Meditation  All Meditat*  All Mindful* | 9303 |
| 5 | Yoga | Su Yoga  All Yog* | 2580 |
| 6 | Combined Terms for Intervention Variable | OR 1-5 | 22661 |
| **Row** | **Outcome Variable** | **Search terms:** | **# of hits:** |
| 7 | Immune System | Su Immune System  Su Immunoproteins  Su Cytokines  Su Immune system phenomena | 4270 |
| 8 |  | All Immun*  All Inflamm*  All Leukocyte  All Lymphoycte  All Antibod*  All Cytokine  All C-reactive protein | 62728 |
| 9 | Psychoneuroimmunology | Su Psychoneuroimmunology  All Psychoneuroimmunology | 414460 |
| 10 | Combined Terms for Outcome Variable | 7 or 8 or 9 | 466183 |
| 11 | **Combined Terms for Intervention and Outcome** | **6 AND 10** | **2874** |
| 12 | **Limit to English Language** |  | **2630** |
| 13 | **Limit to Humans** |  | **2449** |
| 14 | **Limit to Scholarly Journals** |  | **1460** |

1. **CINAHL and SPORTDiscus**

| **Row** | **Intervention Variable** | **Search terms:** | **# of hits:** |
| --- | --- | --- | --- |
| 1 | Mind-Body Therapies | All mind-body  All mind body | 3767 |
| 2 | Tai Chi | All Tai Chi  All Tai Chi Chuan  All Tai Chi Chih  All T’ai Chi  All Tai Ji  All Tai Ji Quan  All Taijiquan | 2233 |
| 3 | Qi Gong | All Qi Gong  All Qigong  All Qi-gong  All Qi-training  All Chi Kung | 715 |
| 4 | Meditation | All Meditat*  All Mindful* | 4912 |
| 5 | Yoga | All Yog* | 13676 |
| 6 | Combined Terms for Intervention Variable | OR 1-5 | **22689** |
| **Row** | **Outcome Variable** | **Search terms:** | **# of hits:** |
| 7 | Immune System | All Immun*  All Inflamm*  All Leukocyte  All Lymphoycte  All Antibod*  All Cytokine  All C-reactive protein | 154663 |
| 8 | Psychoneuroimmunology | All Psychoneuroimmunology | 435 |
| 9 | Combined Terms for Outcome Variable | 7 or 8 | 154847 |
| 10 | **Combined Terms for Intervention and Outcome** | **6 AND 9** | **508** |
| 11 | **Limit to English Language** |  | **506** |
| 12 | **Limit to Humans** |  | **302** |
