# Supplementary material for: The Effects of Mind-Body Therapies on the Immune System: Meta-Analysis
Source: PLoS One. 2014 Jul 2;9(7):e100903. doi: 10.1371/journal.pone.0100903 (PMC4079606; doi:10.1371/journal.pone.0100903)
Supplement: Table S2 — Assessment of overall risk of bias*. (DOCX) [file pone.0100903.s002.docx]

**Table S2. Assessment of overall risk of bias***

| **Reference** | **Randomization** | **Allocation Concealment** | **Blinding of Outcome Assessment** | **Drop-out Rate** | **All Randomized Included in Analysis** | **Selective Reporting** | **Overall Risk of Bias** |
| --- | --- | --- | --- | --- | --- | --- | --- |
| Barrett 2012 | Low | Low | Low | 3% | No | High | **Low** |
| Cade 2010 | Unclear | Unclear | Unclear | 17% | No | Low | **Moderate** |
| Chen 2006 | Unclear | Unclear | Unclear | 3% | Unclear | Unclear | **High** |
| Chen 2010 | Low | Unclear | Unclear | 11% | No | Unclear | **Moderate** |
| Creswell 2009 | Unclear | Unclear | Low | 42% | Yes | Low | **Low** |
| Creswell 2012 | Low | Unclear | Low | 15% | Yes | High | **Low** |
| Davidson 2003 | Unclear | Unclear | Unclear | 15% | Unclear | Unclear | **High** |
| Elsenbruch 2005 | Unclear | Unclear | Unclear | 0% | Yes | Unclear | **High** |
| Fan 2010 | Unclear | Unclear | Unclear | 0% | Yes | Unclear | **High** |
| Gopal 2011 | Unclear | Unclear | Unclear | Unclear | Unclear | Unclear | **High** |
| Hidderley 2004 | Low | Unclear | Unclear | Unclear | Unclear | Unclear | **High** |
| Irwin 2003 | Low | Unclear | Low | 14% | Yes | Unclear | **Low** |
| Irwin 2007, 2012 | Low | Unclear | Low | 9% | Yes | No | **Low** |
| Janelsins 2011, Sprod 2012 | Low | Low | Unclear | 32-40% | Yes | Unclear | **Low** |
| Lavertsky 2011 | Low | Low | Low | 7% | Yes | Unclear | **Low** |
| Lengacher, 2011 | Unclear | Unclear | Unclear | 2% | No | Unclear | **High** |
| Malarkey 2013 | Low | Unclear | Low | 9% | Yes | Unclear | **Low** |
| Manzaneque 2004 | Unclear | Unclear | Unclear | 10% | No | Unclear | **High** |
| Manzaneque 2009 | Unclear | Unclear | Low | 15% | No | Unclear | **Moderate** |
| McCain 2008 | Low | Unclear | Low | 19% | Yes | No | **Low** |
| Oh 2008, 2010, 2012 | Low | Unclear | Unclear | 33% | Yes | Unclear | **Moderate** |
| Oken 2010 | Low | Unclear | Low | 13% | No | Unclear | **Low** |
| Pace 2009 | Low | Low | Unclear | 31% | No | Unclear | **High** |
| Pullen 2008 | Unclear | Unclear | Low | 0% | Yes | Unclear | **Moderate** |
| Pullen 2010 | Unclear | Unclear | Low | 15% | Unclear | Unclear | **Moderate** |
| Rao 2008 | Low | Low | Unclear | 30% | No | Unclear | **High** |
| Rosenkranz 2013 | Unclear | Unclear | Unclear | Unclear | Unclear | Unclear | **High** |
| SeyedAlinaghi 2012 | Low | Unclear | Unclear | 30% | No | Unclear | **High** |
| Solberg 1995 | Unclear | Unclear | Unclear | 0% | Yes | Unclear | **High** |
| Subramanian 2012 | Unclear | Unclear | Unclear | 7% | No | Unclear | **High** |
| Taylor 1995 | Unclear | Unclear | Unclear | 0% | Yes | Unclear | **High** |
| Vogler 2011 | Unclear | Unclear | Unclear | 5% | No | Unclear | **High** |
| Wang 2011 | Unclear | Unclear | Unclear | 0% | Yes | Unclear | **High** |
| Zautra 2008 | Low | Unclear | Low | 9% | Yes | Unclear | **Low** |

*****Overall judgment did not include blinding of participants/personnel due to the nature of mind-body intervention studies. Selective reporting most often marked as unclear as protocols not available via clinicaltrials.gov. Attrition bias was judged based on the attrition rate and whether or not all randomized participants were included in the analysis. Specifically, attrition rate was determined by dividing the number of participants who left the study through the final time-point by the total number randomized. If the attrition rate exceeded 20% and only a completion analysis was performed the study was judged as having a high risk of attrition bias. Finally, unclear risk of bias was assumed to be high risk of bias.
